# Interactive programs with preschool children bring smiles and conversation to older adults: time-sampling study

**DOI:** 10.1186/1471-2318-13-111

**Published:** 2013-10-18

**Authors:** Kumiko Morita, Minako Kobayashi

**Affiliations:** 1 Department of Health Education, Graduate School of Health Care Sciences, Tokyo Medical and Dental University, 1-5-45 Yushima, Bunkyo-ku, Tokyo 1138519, Japan; 2 Faculty of Nursing, Kameda College of Health Sciences, 462 Yokosuka, Kamogawa, Chiba 2960001, Japan

**Keywords:** Intergenerational programs, Older adults, Smiles, Conversation, Engagement/behaviour, Time sampling

## Abstract

**Background:**

Keeping older adults healthy and active is an emerging challenge of an aging society. Despite the importance of personal relationships to their health and well-being, changes in family structure have resulted in a lower frequency of intergenerational interactions. Limited studies have been conducted to compare different interaction style of intergenerational interaction. The present study aimed to compare the changes in visual attention, facial expression, engagement/behaviour, and intergenerational conversation in older adults brought about by a performance-based intergenerational (IG) program and a social-oriented IG program to determine a desirable interaction style for older adults.

**Methods:**

The subjects of this study were 25 older adults who participated in intergenerational programs with preschool children aged 5 to 6 years at an adult day care centre in Tokyo. We used time sampling to perform a structured observation study. The 25 older participants of intergenerational programs were divided into two groups based on their interaction style: performance-based IG program (children sing songs and dance) and social-oriented IG program (older adults and children play games together). Based on the 5-minute video observation, we compared changes in visual attention, facial expression, engagement/behaviour, and intergenerational conversation between the performance-based and social-oriented IG programs.

**Results:**

Constructive behaviour and intergenerational conversation were significantly higher in the social-oriented IG programming group than the performance-based IG programming group (p<0.001). No significant differences were observed in frequency of smiles, however, when weighted smiling rate was used, smiles were significantly more frequently observed in the social-oriented IG programming group than the performance-based IG programming (p<0.05). The visual attention occurred between the generations was significantly higher in the performance-based IG programming group than the social-oriented IG programming group (p<0.05).

**Conclusions:**

Intergenerational programs with preschool children brought smiles and conversation to older adults. The social-oriented IG program allowed older adults to play more roles than the performance-based IG program. The intergenerational programs provide opportunities to fulfil basic human needs and reintegrate older adults into society. Further development of such beneficial programs is warranted.

**Trial registration:**

UMIN-CTR clinical Trial: UMIN000010439

## Background

Older adults are at risk of being socially isolated due to poor health, low morale, and communication difficulties [1]. Keeping older adults healthy and active as vital members of their communities is an emerging challenge of our aging society. Despite the importance of personal relationships to health and well-being [2,3], changes in the family structure, including the emergence of nuclear families and a higher divorce rate, have resulted in a lower frequency of intergenerational interactions [4]. Even in countries with traditionally strong family ties, such as Japan, the proportion of three-generation-family households has decreased, while that of aged households has increased from 6.3% in 1986 to 20.5% in 2011 [5]. According to the International Comparison Survey on the Daily Life and Attitudes of Elderly Persons (2010), 51.9% of people aged 60 and over who live separately from their children reported having contact with their children ‘more than once a week’ in Japan, while this rate was approximately 80% in the United States and around 60% in Sweden, Korea and Germany [6].

Intergenerational (IG) programs were proposed as a way to develop bonds between two generations in the United States and have expanded to other regions of the world [4]. As defined by the International Consortium for Intergenerational Programs, “intergenerational programs” are “social vehicles that create purposeful and ongoing exchanges of resources and learning among older and younger generations” [7]. Intergenerational programs provide contact and communication between children and older adults. Communication enables older adults to help others by listening, reflecting and offering advice [8], and serves critical roles in the lives of older adults, including maintaining a sense of identity, and relieving loneliness, depression or anxiety [9]. The literature suggests that intergenerational programs benefit both older adults and children. For older adults, the effects of programs include increased self-esteem, improved well-being [10-12], increased social contact [10], decrease distress [13,14], and gratification for their contribution to the community [15], while positive attitudes towards the elderly [16-19], and understanding of the aging process [11,15] have been reported for children.

The Intergenerational School (TIS) in Ohio in the United States represents a model of intergenerational programs in which older adults in the community teach reading and mathematics to children as mentors [20]. In Japan, it was observed that healthy older adults can lead the interaction (e.g., help children with their studies and teach regional culture [21], and also read picture books to children [22]. These elder-led programs, which motivate older adults to participate in society, are still few. According to a recent study of adult day services in Tokyo, more than 80% of the day centres reported that the frequency of intergenerational programs with elementary school children was “a few time a year” [23].

The IG programs in current elder day services in Japan include a “performance-based IG program” (children sing songs and dance) and a “social-oriented IG program” (older adults and children play games together). Depending on the available exchange time, either program or a combination of both programs is implemented. The performance-based and social-oriented IG programs each have advantages and disadvantages. In the performance-based IG program, even physically vulnerable older adults (e.g., those using a wheel chair) can participate in the program. However, this program does not promote the older adults’ autonomy because the older adults are passive [24]. The social-oriented IG program generates conversation among individuals with different generations. However, if children do not show interest in the social-oriented IG program, fewer conversations and mutual exchanges occur between the children and older adults [25]. Interaction programs should be meaningful for both older adults and children, but there are few studies that have examined the differences between different interaction styles.

The present study aimed to compare the changes in visual attention, facial expression, engagement/behaviour, and intergenerational conversation in older participants in performance-based and social-oriented IG programs to determine a desirable interaction style for older adults.

## Methods

### Design

Using time sampling, we conducted a structured observation study, which was used to document specific behaviours, actions, and events [26]. Time sampling is a method in which a designated amount of time (observational unit) is set and behaviour is observed within this period [27].

### Participants and setting

The participants of this study were 25 older adults aged 71 to 101 years who participated in intergenerational programs with preschool children aged 5 to 6 years (one group consisted of about 20 children) at a day care centre in Tokyo and for whom video observation was available. Regarding the Activities of Daily Living (ADLs), the participants were independent or could walk with walking assistance devices. Regarding cognitive function, we used the day-service user information under long-term insurance. The level of independent living in the elderly is considered from the aspect of cognitive function in addition to physical function to determine eligibility for long-term care insurance using the national criteria [28]. The levels of independent living in the national criteria (independent, I, IIa, IIb, IIIa, IIIb, IV, M), range from “independent” to “requirement of specialty mental care due to severe mental health problems such as delusion and harm to self or other (M).” In the present study, we included the elderly in the levels of “independent,” “mostly independent (I),” and “some noticeable mistakes in activities outside home but fairly well at home with supervision (IIa),” and excluded those who required assistance with their daily activities due to symptoms of severe cognitive impairment (IIb, IIIa, IIIb, IV, M) from the study.

The participating day care centre was founded in 1987, and has provided programs to promote communication with community residents. Children from a nursery school or kindergarten in the neighbourhood visit older adults at the day care centre once or twice a month and spend 20 – 30 minutes with them at each visit.

### Data collection

The study took place from December 2011 to February 2012. Three intergenerational programs during the study period were recorded with two digital high-vision cameras (Panasonic, HDC-TM90). The first program (December 2011) was follows: The “performance-based IG program” included 11 older adults for observation. Each child answered questions about their activities on Christmas Day at home, while older adults sat in chairs and listened to the children talk. The second program (January 2012): The “social-oriented IG program” included 8 older adults for observation. Children and older adults were divided into three groups, and they played “Karuta (a traditional Japanese playing card game),” “Cat’s cradle,” and “Fuku-warai (a game similar to ‘pin the tail on the donkey’)” together. The third program (February 2012): The “social-oriented IG program” included 6 older adults for observation. One elder and 3 to 4 children played “Action Songs” (hand play) as one group.

### Measurement

We assessed the changes in visual attention, facial expression, engagement/behaviour, and intergenerational conversation among the participants during the intergenerational programs. According to the Japanese version of the Intergenerational Exchanges Attitude Scale (IEAS) [29], we developed an observation record form (Table 1). The inter-rater reliability of IEAS was established based on a kappa coefficient (0.60~0.90). We used the same ratings for ‘change in visual attention’ and ‘facial expression’ (positive, neutral, and negative) as in IEAS. Visual attention had same generation (the elderly attention was directed to the same generation), intergeneration (the elderly attention was directed to children) and other categories, and each was a dichotomous variable (yes vs. no). Regarding facial expression, because laughter uses the most facial muscles and the brain is more stimulated following laughing and smiling [30,31], we weighted positive facial expression in three phases as follows based on the facial expression analysis of Ekman et al. [32]:

**Table 1:** The example of the observation form

**Unit**		**Visual attention**			**Facial expression**					**Engagement/behaviour**		**Intergenerational conversation**	
		**Same generation**	**Inter-generation**	**Others**	**Positive**			**Neutral**	**Negative**	**Constructive**	**Passive**	**Yes**	**No**
					**Smile 3**	**Smile 2**	**Smile 1**	**No expression**	**Anger/antipathy**				
1	0:00^~^		**√**			**√**				**√**			**√**
2	0:15^~^		**√**		**√**					**√**		**√**	
3	0:30^~^	**√**					**√**				**√**		**√**
4	0:45^~^		**√**			**√**					**√**		**√**
5	1:00^~^		**√**					**√**			**√**		**√**
▪	▪												
▪	▪												
▪	▪												
20	4:45^~^		**√**		**√**					**√**		**√**	
	Total	4	16	0	4	8	6	2	0	12	8	7	13

Smile 1= smiling/interest with change in mouth angle and eyes only. Smile 2=smiling with mouth open indicating joy or surprise. Smile 3 = laughter, change in eyes with vocalization to accompany smile.

We assessed ‘engagement/behaviour’ in accordance with the Myers Research Institute Engagement Scale (MRI-ES) as developed by Judge et al. [33]. Engagement/behaviour had constructive (the elderly positively participated in the program) and passive (the elderly participated in the program but only watching and listening without voluntary behaviours) engagement categories, and each was a dichotomous variable (yes vs. no). We observed ‘Intergenerational conversation’ which was measured by yes or no. When the elderly talked to children, we checked “yes” in intergeneration conversation. We counted conversation only between the older adult and the child, and did not include conversation between individuals of the same generation.

The observation time was determined to be 5 minutes (a total of 20 units), with one unit consisting of a 15-second interval as proposed by Nakazawa et al. [27] and Matsuura et al. [34]. The 5-minute observation was the same as in the revised version of Elder-Child Interaction Analysis developed by Newman et al. [35] for the behavioural scale of intergenerational interactions and IEAS. A 5-minute video observation was conducted in the middle of the program in which stable interactions could be observed. We observed changes in visual attention, facial expression, engagement/behaviour, and intergenerational conversation every 15 seconds, and put a check mark on the observation form. When more than two categories were observed during the same unit with regard to changes in visual attention and engagement/behaviour, only the longest observed category was marked with a check. Regarding positive facial expression, when multiple categories were observed during the same unit, we marked the larger expression with a check (e.g., both laughter and smile). Based on the 5-minute video observation, we compared changes in visual attention, facial expression, engagement/behaviour, and intergenerational conversation between the child-led and interactive interaction programs using the indicators in Table 2.

**Table 2:** Indicators

	**Formula**	
Visual attention occured between the generations =	Number of intergenerational attention units/	×100
	Observational time 20 units (5 min.)	
Smiling rate =	Number of positive units/	×100
	Observational time 20 units (5 min.)	
Weighted smiling rate =	(Number of Smile 3 units × 3 + Number of Smile 2 units × 2 + Number of Smile 1 units)/	×100
	Observational time 20 units (5 min.)	
Constructive behaviour rate =	Number of constructive units/	×100
	Observational time 20 units (5 min.)	
Intergenerational conversation rate =	Number of intergenerational conversation units/	×100
	Observational time 20 units (5 min.)	

### Data analysis

The 25 older participants in intergenerational programs were divided into two groups by interaction style. Eleven older adults who participated in the first program were in the performance-based IG programming group, while 14 older adults who participated in the second and third programs were in the social-oriented IG programming group. We used Pearson’s χ^2^test and the Mann–Whitney U test. A P-value of <0.05 was considered to be statistically significant.

### Ethical considerations

All the participants were given full oral and written information about the study, and they all provided written informed consent before the study. For preschool children, the principal of the nursery school or kindergarten was fully informed of the study, and written information about the study was given to their parents by the principal. We obtained informed consent for the study and also signed written consent from their parents for video recording. The confidentiality of personal data was preserved. This study was approved by the Ethical Review Board of the Faculty of Medicine at Tokyo Medical and Dental University (No. 1079). This trial has been registered with UMIN-CTR clinical trial (UMIN000010439).

## Results

### Participant characteristics

Table 3 shows the characteristics of the participants. Of 25 participants, there were 5 males (20.0%) and 20 females (80.0%), and the mean age was 85.0±7.5 years old. Eight older adults (32.0%) lived alone. There were 14 older adults without cognitive impairment (56.0%). The mean frequency of day service use was 2.0±1.0/week. There were no significant differences in characteristics between the performance-based and social-oriented IG programming groups.

**Table 3:** Participants' characteristics

		**Total (n=25)**	**Performance-based IG programming group (n=11)**	**Social-oriented IG programming group (n=14)**	**Significance**
		**n (%)**	**n (%)**	**n (%)**	
Sex	Male	5 (20.0)	2 (18.2)	3 (21.4)	
	Female	20 (80.0)	9 (81.8)	11 (78.6)	n.s. ※1
Mean age (years)		85.0±7.5	83.0±5.8	86.6±8.6	n.s. ※2
Living	Alone	8 (32.0)	3 (27.3)	5 (35.7)	
	With family	16 (64.0)	8 (72.7)	8 (57.1)	n.s. ※1
	Unknown	1 (4.0)	0 (0.0)	1 (7.1)	
Cognitive	Independent	14 (56.0)	6 (54.5)	8 (57.1)	
Function	I or IIa※3	11 (44.0)	5 (45.5)	6 (42.9)	n.s. ※1
Use of day service (Mean number of use/week)		2.0±1.0	1.9±0.7	2.2±1.1	n.s. ※2

※1 χ^2^ test.

※2 Mann–Whitney U test.

※3 Level of Cognitive Function (by MHLW).

### Differences by interaction style

Kappa coefficients among the 2 reviewers were assessed for 6 participants (25% of all the subjects). Lombard et al. [36] recommended 10% as the minimally, acceptable subsample to use for interrater reliability coding. The kappa coefficients of change in visual attention, facial expression, engagement/behaviour, and conversation were 0.82, 0.80, 0.76, and 0.86, respectively.

Constructive behaviour and intergenarational conversation were significantly higher in the social-oriented IG programming group than the performance-based IG programming group (p<0.001). No significant differences were observed in smiles, however, when weighted smiling rate was used, smiles were significantly more frequently observed in the social-oriented IG programming group than the performance-based IG programming group (p<0.05). The visual attention occured between the generations was significantly higher in the performance-based IG programming group than the social-oriented IG programming group (p<0.05) (Table 4).

**Table 4:** Comparison by the interaction style

	**Interaction style**	**Median**	**Mean rank**	**Significance level**
Visual attention occurred between the generations	Performance-based	100.0	16.68	0.025
(0~100)	Social-oriented	87.5	10.11	
Smiling rate	Performance-based	45.0	10.59	0.149
(0~100)	Social-oriented	80.0	14.89	
Weighted smiling rate	Performance-based	55.0	9.36	0.029
(0~300)	Social-oriented	115.8	15.86	
Constructive behaviour rate	Performance-based	0.0	6.00	0.000
(0~100)	Social-oriented	90.0	18.50	
Intergenerational conversation rate	Performance-based	0.0	6.50	0.000
(0~100)	Social-oriented	17.5	18.11	

Mann–Whitney U test.

## Discussion

In the present study, there were differences in the changes in visual attention, facial expression, engagement/behaviour and intergenerational conversation in association with the interaction style of intergenerational program. Smiles, constructive behaviour and intergenarational conversation were significantly higher in the social-oriented IG programming group than the performance-based IG programming group, while the visual attention occured between the generations was significantly higher in the performance-based IG programming group than the social-oriented IG programming group.

Smiles were observed in both the performance-based and social-oriented IG programming groups in this study. The older adults smiled just as a result of looking at the children’s faces in the performance-based IG program, while laughter was also heard in the social-oriented IG program, when the older adult picked up a good card, achieved a successful string figure, created a funny face in Fuku-warai, or played paper, rock and scissors. In general, social smiles sometimes occur, but laughter often breaks out when an individual is really enjoying something.

Laughter and smiles are means of non-verbal communication that express interpersonal attitudes. Laughter and smiling are usually produced as messages of good will to others, signalling acceptance. Laughter is believed to have evolved in humans to express a secure and safe message to others [30]. A chain reaction of smiling is known to occur in mother-child interaction when the mother smiles in response to the infant’s smile as a positive feedback [31]. Smiles and laughter allow both children and older adults to feel secure and connected, and help build a positive relationship even in a short interaction. Previous studies reported that following interpersonal contact, older adults showed more positive attitudes toward younger people [16], and also desired a meaningful relationship with younger people [18].

Effective intergenerational programs provide opportunities to plan and reflect on experiences [37]. Although little development can be expected after the completion of a performance in the performance-based IG program, one activity may trigger various conversation topics in the social-oriented IG program as shown by the results of the present study. Active engagement is found to create more opportunities to find and share common interests during the time that older adults and children spent together [37]. In the present study, the constructive behaviour rate was very high (90%) in the social-oriented IG program, and both older adults and children engaged in conversation and enjoyed traditional play together. Both generation groups have much to give and learn through interaction; children have a zest for learning, while older adults have a lifetime of experience [19].

When older adults are given meaningful roles such as the opportunity to nurture and mentor children, their self-esteem increases [11] in association with feeling needed, valued, and a sense of self-worth [10], and older adults are reminded of their role in society. In the present study, older adults not only responded to children’s questions, but also shared knowledge with children by teaching the rules of games and passing on cultural traditions though play. Traditional games invite conversation. Since Karuta and Fuku-warai are often played around the New Year holiday, playing these games presents a good opportunity for the adults to explain the cultural traditions engaged in at New Year’s. The older adults also learned new ways to play from the children regarding the “Cat’s cradle” and “Action Songs.” An intergenerational program is only effective when it supports mutually beneficial interactions [38].

Visual attention is also a form of non-verbal communication [39,40]. In the performance-based IG programming group, older adults looked at children throughout the 5-minute observation period. Although these performance-based IG programs are adopted in a number of day services, the goal of intergenerational interaction is difficult to achieve with only the quiet watching of a performance [41]. It is rather note-worthy that the visual attention occurred between the generations was 87.5% in the present social-oriented IG program.

Close interaction and repeated contact make self-disclosure and other friendship-developing mechanisms possible [42]. It is very difficult to interpret the present findings without knowing whether the older adults and children had regular opportunities to interact with each other. The children participating in this study had visited the day centre on a regular basis since April 2011, and they were thus familiar faces among the older adults at the time of the survey in the winter of 2011. This sense of intimacy may have contributed to the generation of smiles in the results.

Intergenerational programs have the great potential to promote health and well-being of older adults. Given the limited number of such programs at present, we need to develop new programs which attract the participation of both older adults and children, with natural smiling and laughter. There are a variety of potential interactions including the pairing of older adults with children. Since the interest taken by older adults in activities is affected by their experience and character [43], programs utilizing their unique capacities and experience are desirable. For example, an older adult who has been a farmer can be a teacher of horticultural activity, while those who have a good knowledge of plants can be guides for children’s outdoor activities and those who are good at drawing can teach children to draw.

It is important for facilitators to assess whether older adults communicate well with children in the program and to provide support. It is the role of facilitators to offer a program which draws out the strengths of both generations and to promote sustained attention and self-motivated involvement [37], while ensuring that older adults and children are always the main focus of the intergenerational program. Future plans for intergenerational programs should be more research-based, and the principles of contact theory (support from authority, common goals, cooperation, equal group status, and opportunity for friendship) are essential for intergenerational programs [41,42]. Future research – practice interactions may generate successful programs.

### Strengths and limitations

Since the present study was a cross-sectional research design to compare a single set of observations of different adults in intergenerational programs, the effect of continuity of the IG programs was not determined. In addition, the participants in the performance-based IG program and the social-oriented IG program were different, and thus the effect of subject characteristics (e.g., how to express one’s emotion) cannot be ruled out. To eliminate the effect of subject characteristics, the same persons should participate in both the performance-based and social-oriented IG programs in a cross-over research approach. Also, we need to determine whether the same effects can be expected for older adults with severe dementia and whether the contents of intergenerational programs involving elementary school or junior high school children should be the same as those involving preschool children aged 5 to 6. The present study was conducted in one facility with small number of subjects. Therefore we need to increase number of facilities and subjects and examine whether the same results are obtained. Also, randomization of subjects to different interaction-style programs is necessary.

Although data collection is often difficult using questionnaire surveys when the subjects are older adults or preschool children due to problems with the reliability and validity of the survey results, we overcame such difficulties with objective video observation in the present study. Facial expression changed for only a few seconds at a time, therefore it was difficult to observe all of the changes in a single interaction, however, repeated play enabled us to improve the accuracy of the data.

## Conclusions

Intergenerational programs with preschool children bring smiles and conversation to older adults. Smiles and conversation correspond to interpersonal acceptance, which is a basic human need. The social-oriented IG program allows older adults to engage in more roles than the performance-based IG program. When older adults are given meaningful roles as mentors or role models, they are reminded of their ability to contribute to society. Intergenerational programs provide the opportunity to fulfil basic human needs and reintegrate older adults into society. Further development of beneficial programs is warranted.

## Competing interests

The authors declare that they have no competing interests.

## Authors’ contributions

KM was responsible for the study conception and design, data analysis, and manuscript drafting. MK participated in the study conception and design, and advised of the analysis. All authors read and approved the final manuscript.

## Pre-publication history

The pre-publication history for this paper can be accessed here:

http://www.biomedcentral.com/1471-2318/13/111/prepub
